# Modulation of the Mesenchymal Stem Cell Secretome Using Computer-Controlled Bioreactors: Impact on Neuronal Cell Proliferation, Survival and Differentiation

**DOI:** 10.1038/srep27791

**Published:** 2016-06-15

**Authors:** Fábio G. Teixeira, Krishna M. Panchalingam, Rita Assunção-Silva, Sofia C. Serra, Bárbara Mendes-Pinheiro, Patrícia Patrício, Sunghoon Jung, Sandra I. Anjo, Bruno Manadas, Luísa Pinto, Nuno Sousa, Leo A. Behie, António J. Salgado

**Affiliations:** 1 Life and Health Sciences Research Institute (ICVS), School of Health Sciences, University of Minho, Braga, Portugal; 2 ICVS/3B’s, PT Government Associate Lab, Braga/Guimarães, Portugal; 3 Pharmaceutical Production Research Facility (PPRF), Schulich School of Engineering, University of Calgary, Calgary, Alberta, Canada; 4 CNC - Center for Neuroscience and Cell Biology, University of Coimbra, Portugal; 5 Faculty of Sciences and Technology, University of Coimbra, Portugal; 6 Biocant - Biotechnology Innovation Center, Cantanhede, Portugal

## Abstract

In recent years it has been shown that the therapeutic benefits of human mesenchymal stem/stromal cells (hMSCs) in the Central Nervous System (CNS) are mainly attributed to their secretome. The implementation of computer-controlled suspension bioreactors has shown to be a viable route for the expansion of these cells to large numbers. As hMSCs actively respond to their culture environment, there is the hypothesis that one can modulate its secretome through their use. Herein, we present data indicating that the use of computer-controlled suspension bioreactors enhanced the neuroregulatory profile of hMSCs secretome. Indeed, higher levels of *in vitro* neuronal differentiation and NOTCH1 expression in human neural progenitor cells (hNPCs) were observed when these cells were incubated with the secretome of dynamically cultured hMSCs. A similar trend was also observed in the hippocampal dentate gyrus (DG) of rat brains where, upon injection, an enhanced neuronal and astrocytic survival and differentiation, was observed. Proteomic analysis also revealed that the dynamic culturing of hMSCs increased the secretion of several neuroregulatory molecules and miRNAs present in hMSCs secretome. In summary, the appropriate use of dynamic culture conditions can represent an important asset for the development of future neuro-regenerative strategies involving the use of hMSCs secretome.

Human mesenchymal stem/stromal cells (hMSCs) are of great interest in the field of regenerative medicine. Their therapeutic properties can be mainly attributed to their secretome, which has been shown to modulate several processes *in vitro* and *in vivo*, such as cell proliferation, survival, differentiation, immunomodulation, anti-apoptosis, angiogenesis and stimulation of tissue adjacent cells^1^. In fact it has been suggested that using their secretome may open future therapeutic options for cell-free based therapies^2^. Moreover, it has been demonstrated that hMSCs are able to secrete important neuroregulatory molecules such as brain-derived neurotrophic factor (BDNF), nerve growth factor (NGF), insulin growth factor 1 (IGF-1), hepatocyte growth factor (HGF), vascular endothelial growth factor (VEGF), transforming growth factor beta (TGF-β), glial-derived neurotrophic factor (GDNF), fibroblast growth factor 2 (FGF-2), stem cell factor (SCF), granulocyte colony-stimulating factor (G-CSF) and stromal cell-derived factor (SDF-1) both *in vitro* and *in vivo*^1^. In addition to these paracrine soluble factors, recent findings on the hMSC secretome also suggest that its vesicular fraction plays an important role in mediating the processes referred to above^3^. Indeed, it has been shown that hMSCs are able to secrete large amounts of vesicles (microvesicles, exosomes containing not only proteins, but also genetic information such as miRNAs), either constitutively or after activation of signals^3^, of value for a wide panel of diseases^4^,^5^,^6^.

Typically the most utilized source of hMSCs is the bone marrow. However, due to the low frequency of hMSCs in bone marrow it is critical to expand these cells *in vitro* to generate a clinically-relevant number of cells for clinical applications^7^. Conventionally, hMSCs are expanded using static culture flasks in the presence of fetal bovine serum (FBS) or human-sourced supplements. However, these expansion platforms can lead to variable culture conditions (i.e. ill-defined medium components, heterogeneous culture environment and limited growth surface area per volume) and thus are not ideal to meet the expected future demand of quality-assured therapeutic cells for wide implementation of hMSC-related therapies. Previous studies from our group revealed that the use of a serum-free medium condition (e.g. PPRF-msc6) was able to support the rapid and efficient isolation and expansion of hMSCs from different sources^8^,^9^. In addition to developing a well-defined medium, we have also developed a scalable, computer-controlled stirred suspension bioreactor-based microcarrier-mediated bioprocess that can be translated to operate in a closed system^9^. Using stirred suspension bioreactors, a number of advantages can be achieved including: (1) a large number of cells can be expanded in one vessel (minimizing vessel-to-vessel variability and minimizing cost related to labor and consumables), (2) the bioreactors can be operated in a fed-batch or perfusion mode of operation (removing metabolites and inhibitory factors while replenishing growth factors) and (3) the bioreactors can be set up with computer-controlled online monitoring instruments to ensure tight control of process variables such as pH, temperature and dissolved oxygen concentration. Additionally, it has been shown that hMSCs respond to changes in their physiological environment^10^, namely by using dynamic culturing environments, such as those provided by bioreactors^10^,^11^. Therefore it is possible to hypothesize that the modulation, and further enrichment of growth factors/vesicles, of their secretome could be achieved by using these dynamic culturing systems. With this in mind, in the present work we aimed to characterize and analyze the effects of the human bone marrow-derived MSCs (hBM-MSCs) secretome collected from dynamic culture conditions (i.e. suspension bioreactors) to that obtained from standard culture conditions (i.e. static culture flasks).

## Results

### Expansion of hBM-MSCs in Static and Bioreactor Conditions

**Figure 1:**
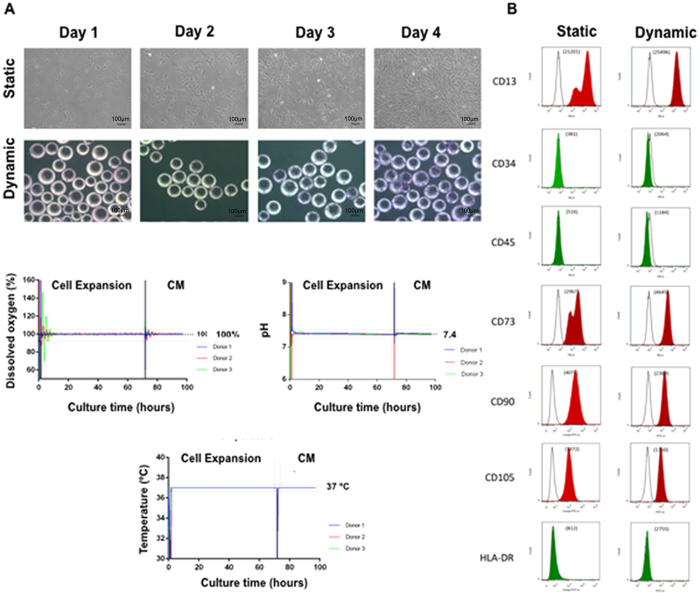
Expansion and characterization of hBM-MSCs in static culture and 500 mL computer-controlled bioreactors. hBM-MSCs adhered to both the tissue culture flasks and the microcarriers in the suspension bioreactors on day 1, and proliferated up to day 4 (**A**). FACS analysis for hMSC markers, CD13, CD73, CD90, CD105 were >99.9% while non-hMSC markers were expressed <2.0%. Mean fluorescence intensity is also displayed (**B**). Additionally, key process parameters (i.e. dissolved oxygen, pH and temperature) in our computer-controlled bioreactor system were well maintained at pre-determined setpoints for the culture period (C). Scale bars represent 100 μm.

We have shown previously that by utilizing a serum-free medium, PPRF-msc6, we can rapidly expand BM-MSCs, compared to using conventional growth medium (i.e. 10% FBS-DMEM)^8^,^9^,^12^. We report herein that, using PPRF-msc6, we were able to rapidly expand cells in both static cultures as well in our 500 mL suspension bioreactors (dynamic culture) (Fig. 1A). The doubling time (i.e. during the exponential growth phase) of the hBM-MSCs in static culture was 37.8 ± 6.0 h, which was similar to the doubling time in dynamic culture (36.4 ± 4.9 h). Additionally, flow cytometry analysis of static and dynamic culture expanded hBM-MSCs revealed that both types of cells expressed the standard hMSCs markers CD13, CD73, CD90 and CD105 at >99.9% and was negative (<2.0%) for CD34, CD45 and HLA-DR (Fig. 1B). In the dynamic bioreactor environment, the dissolved oxygen, pH and temperature were well controlled within the preset set points during the expansion phase and the CM collection phase for all three hBM-MSC donors as noted in Fig. 1C. Cell viability analysis using a Vi-Cell XR Cell Viability Analyzer (Beckman Coulter, Danvers, MA, USA) revealed a greater than 96% cell viability for both conditions.

### The secretome of hBM-MSCs induces neuronal differentiation of hNPCs *in vitro*

**Figure 2:**
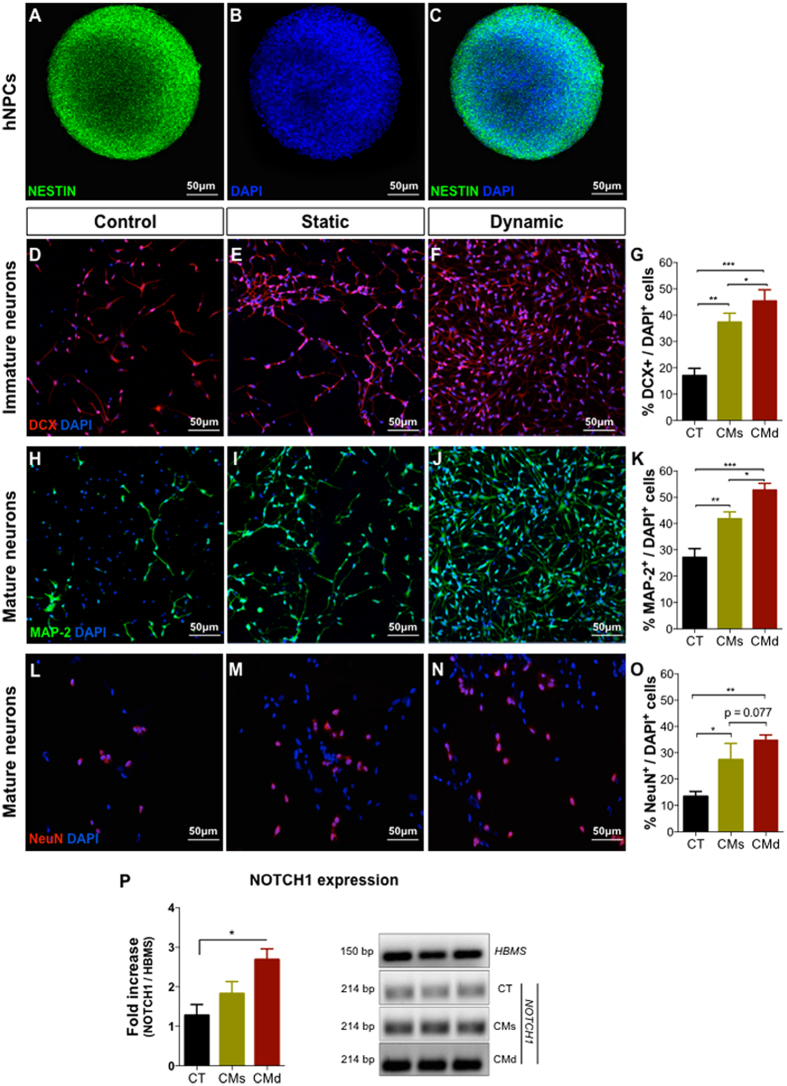
*In vitro* differentiation of hNPCs. Morphology and immunofluorescence staining of undifferentiated hNPCs ((**A–C**): a neuroshpere) into PPRF-h2 showed that the majority of the cells express mainly (**A**) Nestin^+^ cells, which evidences their progenitor profile. hBM-MSCs CM collected from static and dynamic culture conditions was able to significantly increase the survival and differentiation of hNPCs into (**E,F**) immature (DCX^+^ cells) and (**I,J,M,N**) mature (Map-2^+^/NeuN^+^ cells) neurons when compared to the (**D,H,L**) control group ((**G,K,O**); mean ± SEM., n = 3, p < 0.001). At the same time, the (**F,J,N**) hBM-MSCs CM collected from the dynamic culture conditions also showed increased levels of DCX^+^ (p < 0.05) and MAP-2^+^ (p < 0.05) cells, and NeuN^+^ (p = 0.077) cells compared to the (**E,I,M**) static hBM-MSCs CM in the induction hNPC differentiation ((**G,K,O**); mean ± SEM., n = 3). qRT-PCR analysis to NOTCH1 on hNPCs-differentiated cells showed a higher expression (p < 0.05; mean ± SEM; n = 3) in the (**P**) hBM-MSCs dynamic secretome group when compared to the control group. CT: Control (Neurobasal-A media), CMs: hBM-MSCs static conditioned media. CMd: hBM-MSCs dynamic conditioned media (Scale bar: 50 μm). Data are expressed as mean ± SEM. *p<0.05; **p<0.01; ***p<0.001.

hNPCs grew as neurospheres in a serum-free medium PPRF-h2 (Fig. 2A–C) as was already shown^13^. Upon dissociation and plating of the cells in adherent plates with the hMSC CM, the cells adhered and started to differentiate. During the 5 days incubation with the hBM-MSCs CM, cell viability was measured to be above 95% for both conditions. Immunocytochemistry analysis revealed that when hNPCs were incubated for 5 days with the hBM-MSC CM, there was a significant increase in the cell population expressing markers of DCX (immature neurons; F_(2,6)_ = 40.48; p < 0.001; η^2^_partial_ = 0.931 Fig. 2E,F), MAP-2 (mature neurons; F_(2,6)_ = 104.10; p < 0.001; η^2^_partial_ = 0.972); Fig. 2I,J) and NeuN (mature neurons, F_(2, 6)_ = 19,41; p < 0.01, η^2^_partial_ = 0.866; Fig. 2M,N) when compared to control group (incubation with Neurobasal A medium (Fig. 2D,H,L). Additionally it was possible to observe that the CM collected under dynamic culture conditions induced an increased differentiation of hNPCs, as assessed by DCX (p = 0.047), MAP-2 (p = 0.023) and NeuN (p = 0.077) immunostaining, when compared to the group incubated with the CM collected under static conditions (Fig. 2G,K,O). In addition to this, we have concluded that this neuronal differentiation/maturation induction by hBM-MSCs dynamic secretome is regulated through the Notch signaling pathway. Indeed, we observed (on hNPCs-differentiated cultures with dynamic CM) a significant increase of NOTCH1 expression (F_(2,6)_ = 6.213, p = 0.015; η^2^_partial_ = 0.621) when compared to the control group (Neurobasal-A; Fig. 2P).

### The secretome of hBM-MSCs increase the levels of proliferation and induced neuronal differentiation *in vivo*

**Figure 3:**
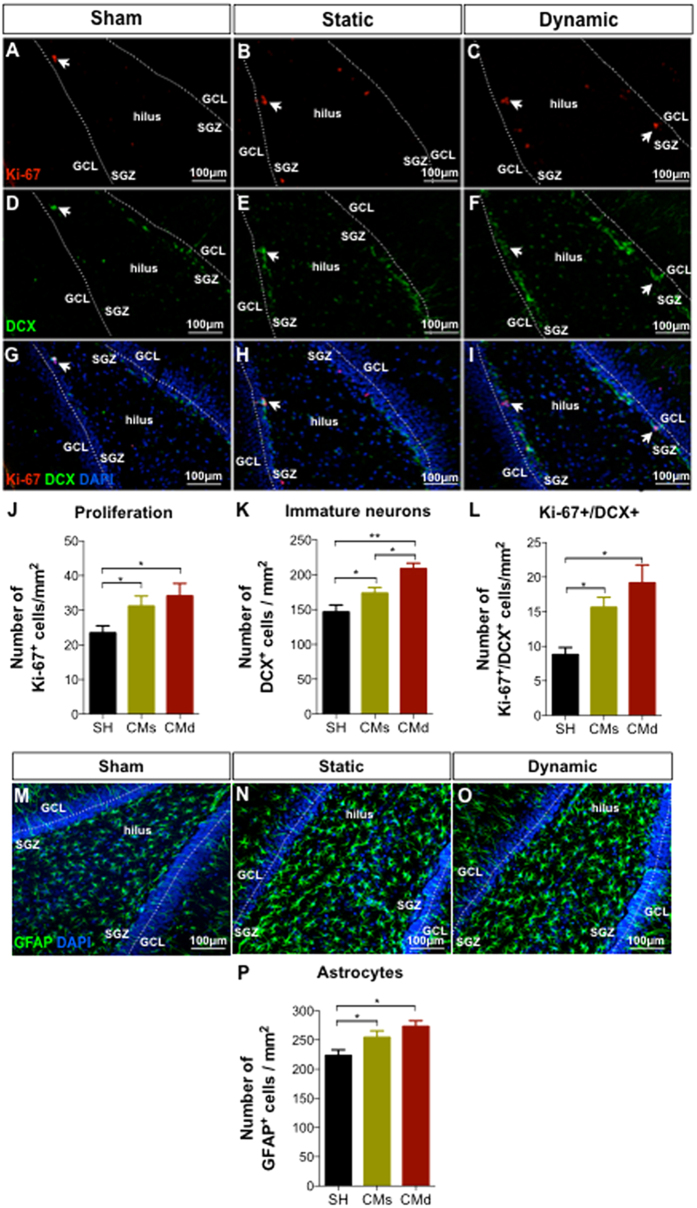
*In vivo* injection of the hMSC Secretome (i.e. CM) increases proliferation and neuronal differentiation. Injection of the hBM-MSC CM from static and dynamic conditions into the DG of adult rat hippocampus. After 7 days post-injection, both CM (static and dynamic) were able to increase the (**B,C**) levels of endogenous proliferating cells (Ki-67^+^ cells) in the DG when compared to the (**A**) sham group ((**J**), mean ± SEM., n = 5, p < 0.05). Moreover, although both CM were able to increase the number of newborn neurons ((**E,F**); DCX^+^ cells) compared to the (**D**) sham group, the dynamic CM showed higher numbers of the newborn (neurons) cell densities ((**K**), mean ± SEM, n = 5, p < 0.05) compared to the static CM. For the induction of neuronal differentiation, both CM (from the static and dynamic conditions) were able to increase significantly the number of Ki-67^+^/DCX^+^ cells when compared to the control group ((**L**), mean ± SEM., n = 5, p < 0.05), with this phenomena being more evident for the group cultured with the dynamic CM. hBM-MSCs CM (from static and dynamic conditions) was also able to increase astrocytic cell densities in the DG of hippocampus. Immunohistochemical analysis of GFAP (Astrocytes; (**M–O**)) revealed increased numbers promoted by the injection of hBM-MSCs CM (**P**, statistically significant to the Sham group, mean ± SEM, n = 5, p < 0.05) 7 days post-injection. SH: Sham (animals injected with Neurobasal-A media) CMs: hBM-MSCs static conditioned media. CMd: hBM-MSCs dynamic conditioned media (Scale bar: 100 μm). Data are expressed as mean ± SEM. *p<0.05; **p<0.01.

To assess the role of hBM-MSCs CM from static and dynamic conditions *in vivo*, animals were bilaterally injected (0.5 μL of CM) into the hippocampal DG without any concomitant immunosuppression therapy. To access proliferation, Ki-67 marker was used. Most of the proliferating cells (Ki-67^+^) were found mainly in the SGZ of the hippocampus (Fig. 3A–C). Moreover, the analysis of the cell proliferation in the DG, revealed a significant increase in the number of Ki-67^+^ cells within the CM-injected DG (F_(2,12)_ = 6.67; p = 0.011; η^2^_partial_ = 0.526; Fig. 3B,C,J) when compared to the control group (Sham; Fig. 3A).

After observing that both CM (from the static and dynamic conditions) were able to stimulate cell proliferation in the DG, we next aimed to determine their effects on the differentiation of DG resident cells. Seven days post-injection of both CM (Fig. 3D–F), we observed an increase in the number of DCX-expressing cells (newborn neurons) in all DG granular layers (F_(2,12)_ = 31.87; p < 0.01; η^2^_partial_ = 0.842), namely in the SGZ (Fig. 3E,F,K) when compared to the Sham group (Fig. 3D). However, injection of the dynamic CM (Fig. 3F) resulted in a significantly higher number of DCX-expressing cells (p = 0.016) when compared to the static-CM group (Fig. 3E,K). After analyzing the co-expression of Ki-67^+^ and DCX^+^ cells, an indication of neuronal differentiation, it was observed that both CM were able to significantly increase (F_(2,12)_ = 12.64; p < 0.05; η^2^_partial_ = 0.678) the number of differentiating neurons when compared to the Sham group (Fig. 3G–I,L). At the same time, although without statistical differences, the dynamic CM presented a positive trend when compared to the static CM (Fig. 3L). Similar effects were also obtained for astrocytic cells (Fig. 3M–O), in which, both CM were able to significantly increase GFAP-positive cell densities (F_(2,12)_ = 6.902; p = 0.010; η^2^_partial_ = 0.535; Fig. 3P) when compared to the Sham group.

### hBM-MSCs secretome proteomic analysis

**Figure 4:**
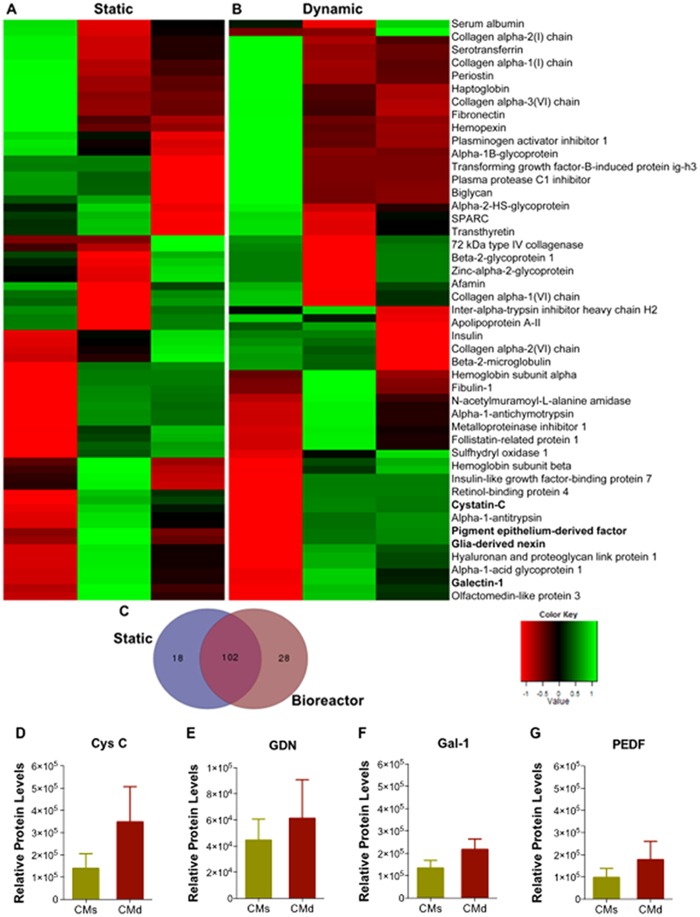
Graphical representation of the hBM-MSCs Secretome (i.e. CM) proteomic analysis by mass spectrometry. CM analysis shows that the pattern of protein expression is modulated when we change from (**A**) static to (**B**) dynamic culture condition. Indeed, the (**C**) Venn diagram indicates more proteins were identified in the dynamic CM (130 proteins) when compared do the static CM (120 proteins). Specific neuroregulatory molecules such as (**D**) cystatin C (Cys C), (**E**) glia-derived nexin (GDN), (**F**) galectin-1 (Gal-1) and (**G**) pigment epithelium-derived factor (PEDF) were found to be upregulated in the dynamic hBM-MSCs secretome (data are expressed as mean ± SEM, n=3). CMs: hBM-MSCs static conditioned media. CMd: hBM-MSCs dynamic conditioned media.

**Figure 5:**
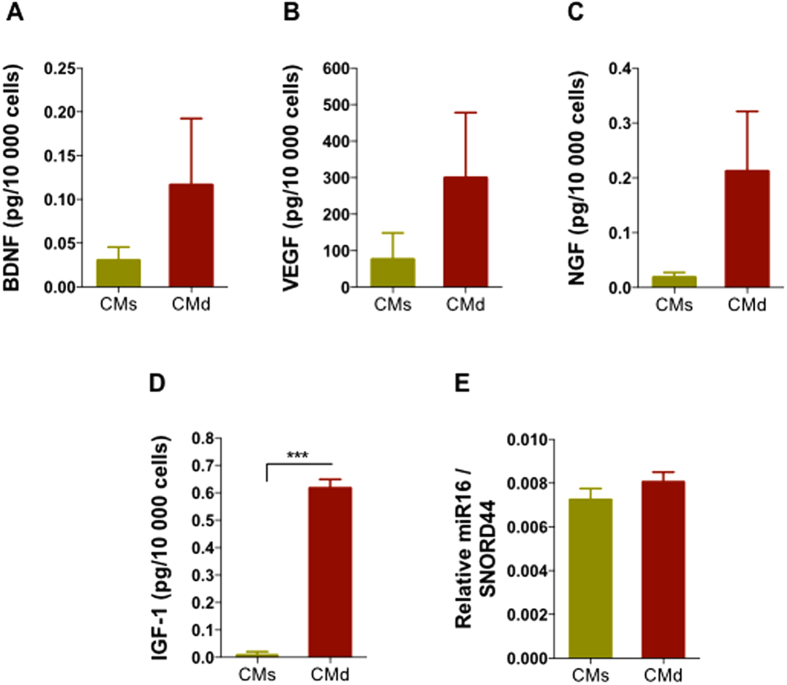
Graphical representation for the hBM-MSCs Secretome (i.e. CM) classical trophic factors by Bioplex analysis. Comparative analysis showed an upregulation in the concentration levels of (**A**) brain-derived neurotrophic factor (BDNF), (**B**) vascular endothelial growth factor (VEGF), (**C**) nerve growth factor (NGF), (**D**) insulin growth factor 1 (IGF-1) and (**E**) miR-16 in the hMSC dynamic CM when compared to the static condition. Trophic factor values were normalized to cell density (i.e. pg (of each factor)) per 10,000 cells in each condition, respectively. CMs: hBM-MSCs static conditioned media. CMd: hBM-MSCs dynamic conditioned media. Values are shown as mean ± SEM, n = 3; ***p < 0.001.

In order to further understand the differences that were evidenced in the *in vitro* and *in vivo* studies, hBM-MSCs secretome (from both the static and dynamic conditions) was characterized through a targeted and non-targeted proteomic approach based analysis namely, Mass Spectrometry (MS/SWATH Acquisition) and Bioplex assays. From the MS/SWATH analysis we observed that the bioreactor-based bioprocess (dynamic condition) modulated the hBM-MSCs secretome to produce a different pattern of protein expression when compared to the secretome collected from the static condition (Fig. 4A,B). Indeed, through the use of Venn diagrams, we were able to identify 120 proteins in the static and 130 proteins in the dynamic condition, in which 102 proteins were common to the two conditions (Fig. 4C). From these, when we analyzed the relative protein levels of the two hBM-MSC secretomes for specific proteins with actions in CNS physiology, we were able to find that molecules (according with the UniProtKB/Swiss-Prot classification) such as cystatin-C (Cys C (P01034); t = 1.211, p = 0.292), glia-derived nexin (GDN (P07093); t = 0.492, p = 0.648), galectin-1 (Gal-1 (P09382); t = 1.397, p = 0.234), and pigment epithelium-derived factor (PEDF (P36955); t = 0.857, p = 0.439), were upregulated in the dynamic conditions (Fig. 4D–G). Moreover it was also found that some other proteins with important roles in CNS regulations were only found in the CM from hBM-MSCs cultured in the bioreactor, namely Ezrin (P15311), Radixin (P35241), Beta-1,4-galactosyltransferase (P15291) and connective tissue growth factor (CTGF; P29279). In line with these findings, the same trend was also observed in the Bioplex based analysis to the hBM-MSCs dynamic secretome concerning the expression of classical trophic factors such as BDNF (t = 1.926, p = 0.126), VEGF (t = 1.995, p = 0.110), NGF (t = 3.055, 0.091), and IGF-1 (t = 31,78, p < 0.001), which were found to be upregulated when compared to the static hBM-MSCs secretome (Fig. 5A–D). In addition to the above proteomic-based analysis, the segregation of miRNAs was also screened by RT-PCR, and miR-16 was found to be upregulated in the CM of dynamic culture conditions (Fig. 5E).

## Discussion

The use of bioreactors has been suggested as a promising alternative to conventional static culture flasks for hMSC expansion^9^,^14^. So far, different bioreactors have been used for hMSC expansion, including fixed bed bioreactors, perfusion bioreactors, and the cultivation of MSCs as aggregates or through the use of microcarriers in stirred suspension bioreactor systems^15^. In the present work, we have expanded hBM-MSCs on microcarriers in computer-controlled stirred suspension bioreactor (Fig. 1). This technology allows us to: (1) expand a large number of cells in one vessel, (2) monitor and ensure tight control of process variables such as pH, temperature and dissolved oxygen concentration (Fig. 1C), and (3) develop a process, which allows for scale-up to larger bioreactor systems. Numerous studies have evaluated the use of microcarriers and suspension bioreactors for the expansion of hMSCs^11^,^16^. Using our serum-free medium (PPRF-msc6), we have shown that it is possible to minimize this lag phase while achieving an 18-fold cell expansion in our microcarrier-mediated suspension bioreactors^9^. Herein, we have shown that by expanding the hBM-MSCs in a computer-controlled suspension bioreactor system, similar cell doubling times and expression of hMSC surface antigens (Fig. 1B) can be achieved, when compared to static culture-expanded cells as it was already stated^8^.

The *in vitro* application of the hBM-MSC secretome obtained from static and dynamic conditions revealed that both were able to induce the differentiation of human CNS-derived cells. Indeed, as shown in Fig. 2, when hNPCs were incubated with the two CM an increased differentiation of hNPCs to the neuronal lineages – both immature (DCX-positive cells; Fig. 2E,F) and mature (MAP-2 and NeuN positive cells; Fig. 2I,J,M,N) neurons - was observed compared to the control group (i.e. Neurobasal-A medium; Fig. 2D,H,L). This is in line to what Sart and colleagues^17^ had already reported regarding the application of hBM-MSC secretome, and the bioactive molecules within it, on NPCs differentiation and maturation. Moreover, they also observed that the hBM-MSCs secretome (just from static conditions) was able to enhance the proliferation, migration and neurite extension of NPCs^17^. In the present study, in addition to these findings, we have also observed that the use of a dynamic hBM-MSCs secretome was more prone in promoting neuronal differentiation/maturation when compared to the secretome collected from static conditions (Fig. 2E–G,I–K,M–O). Based on such evidence, further studies, particular if performed in an injury or disease context, should also focus on trying to access the phenotype and function of newly differentiated neurons. Even though, although the mechanisms by which hMSCs secretome modulates the behavior of neural progenitors remains still unclear, from the molecular point of view, studies have defended that a network of multiple signaling pathways and transcriptional regulators controls the differentiation of neural progenitors^18^. The Notch signaling pathway is one of the most involved pathways in regulating neural progenitors differentiation^19^,^20^,^21^,^22^. In fact, in 2009, Wang and colleagues^23^ demonstrated that MSCs were able to regulate the proliferation and differentiation (e.g. neuronal fate) of neural stem cells (NSCs) through Notch signaling. Indeed, herein, we observed (on differentiated hNPCs cultures) a significant increase of NOTCH1 expression promoted by the hBM-MSCs dynamic secretome (Fig. 2P). Moreover, in addition to their role in controlling precursor cell fate and proliferation, the activation of Notch is involved in neuronal maturation, inducing neurite remodeling and preventing apoptosis^24^.

The *in vivo* experiments revealed that the application of both hBM-MSC CM (dynamic and static) were able to boost cell proliferation within the DG 7 days after their injection, as assessed by the number of Ki-67 positive cells (Fig. 3A–C,J). This was also noted (with BrdU positive cells) in one of our previous works where we compared injection of MSCs isolated from the umbilical cord tissue or their secretome (CM) into the DG^13^. Similar effects were also observed regarding neuronal differentiation. 7 days after the injection of the both secretomes (static and dynamic), it was possible to observe that both of them were able to increase significantly the number of immature neurons (DCX-positive cells) in all the granular layer (particularly in the SGZ) compared to the Sham group (Fig. 3D–F,K). Moreover, we also observed that the dynamic hBM-MSC secretome injected group displayed a significantly increase in neuronal cell densities (DCX^+^ cells) in the DG when compared to the static hBM-MSC secretome injected group (Fig. 3K). Thus, these results indicate that *in vivo*, the secretome of hBM-MSCs acts as a modulator of neural proliferation and differentiation^23^. Actually, the proteomic analysis performed in the present work further revealed that hBM-MSCs produce additional molecules, than those already reported for these type of studies, with a neuroregulatory potential. From our study Cys C, GDN, Gal-1 and PEDF were found to be upregulated in the dynamic conditions (Fig. 4D–G); importantly, these proteins have been reported to have important roles in the migration, differentiation and neuroprotection of neural progenitors and neural cells both *in vitro* and *in vivo*^25^,^26^,^27^,^28^. For instance, Cys C and GDN (upregulated in the dynamic CM, Fig. 4D,E) are known to play crucial roles in the enhancement of neurite outgrowth and neuroprotection through the prevention of oxidative stress^28^,^29^,^30^. Gal-1 and PEDF (upregulated in the dynamic CM, Fig. 4F,G) have been described as important regulators involved in neurogenesis, playing a role on neural stem cells self-renewal and differentiation (trough Notch-dependent pathway) as well as into neuroprotection and functional recovery after the occurrence of CNS disorders^26^,^31^,^32^,^33^. Interestingly, in addition to this, we also identified the presence of 28 specific molecules found only in the dynamic CM (Fig. 4C). From these, we were able to identify more neuregulatory potential molecules such as Ezrin, Radixin, β-1,4-galatosyltransferase and connective tissue growth factor, which have been described as important regulators of neurite outgrowth, neuroregeneration and angiogenesis^34^,^35^,^36^,^37^. Additionally, we have also observed that the use of a dynamic culture condition was also able to increase the concentration levels of well known classical neurotrophic factors such as BDNF, VEGF, and more robustly NGF, and IGF-1 (Fig. 5A–D), which have been described as stronger modulators of neural survival and differentiation^38^,^39^,^40^,^41^,^42^,^43^,^44^,^45^. For instance, BDNF (Fig. 5A) has been described as a booster of neuronal survival and differentiation both *in vitro* and *in vivo*^38^,^39^. VEGF (Fig. 5B) has been described as an activator of divergent intracellular signaling components, able to regulate progenitor cell proliferation and neuronal differentiation^46^. NGF (robustly increased in the dynamic CM, Fig. 5C) is considered to be a strong modulator of neurogenesis as well as, to enhance the survival and neuronal/glial maturation^47^. And finally, IGF-1 (significantly increased in the dynamic CM, Fig. 5D) has been described as an enhancer of neuronal differentiation and hippocampal neurogenesis, as well as an essential component of the signaling network regulating neurogenesis^42^,^44^. Meanwhile, all the above-referred proteins and trophic factors have already been described in the MSCs secretome by different proteomic-based techniques, confirming the results stated by our proteomic analysis^48^,^49^. In addition to the secretion of growth factors and cytokines by MSCs, we also know that they are able to secrete microvesicles and exosomes, which are involved in the transference of genetic material such miRNAs to other cells^1^,^6^. Indeed, from our miRNA-based analysis, we have found that the use of a dynamic culture condition was able to up-regulate the expression of miR-16 (Fig. 5E), which has been described as a potential player in neuronal differentiation^50^, thereby supporting the results reported herein.

Thus, we hypothesize that the modulating effect in neurogenesis triggered by the hBM-MSCs secretome could be related with the increased presence/expression of PEDF, Gal-1, BDNF, VEGF, NGF, IGF-1 and by miRNAs such as miR-16. Our findings suggest that the stimulation of neurogenesis both *in vitro* and *in vivo* by MSCs is not dependent upon the presence of one secreted factor, but several, allowing the gaining of new insights into the release and interplay of these soluble factors/miRNAs and their neurogenic effects, which may lead to a rational design of new therapeutical strategies for the functional recovery of neurological or neurodegenerative disorders.

## Conclusions

In the present work, we have demonstrated that the use of computer-controlled stirred suspension bioreactors improves the neuroregulatory properties of the hMSC secretome. In fact, using CM from dynamically cultured hBM-MSCs (i.e. the secretome), it was possible to induce a significantly higher number of human neural progenitors to differentiate into neurons at different stages of maturation when compared to the hMSC secretome collected under static conditions. Additionally, *in vivo* injections into the hippocampal DG revealed that hBM-MSC dynamic secretome induced neurogenesis, as well as a robust increase in neuronal cell differentiation. These outcomes were associated either with the exclusive presence, or increased expression, of neuroregulatory molecules and miRNAs within the dynamic secretome constitution such as PEDF, Galectin-1, BDNF, VEGF, NGF, IGF-1 and miR-16, which are considered to be important regulators/modulators of the neurogenic and neural differentiation processes. Thus, our results suggest that the use of a dynamic culture condition (i.e. computer-controlled stirred suspension bioreactors) improves the action of the hBM-MSC secretome to modulate neural progenitor proliferation and differentiation, which may open novel therapeutic opportunities in the future.

## Methods

### Expansion of hBM-MSCs in static culture and collection of static conditioned medium

The serum-free medium (PPRF-msc6) used to isolate and expand the hBM-MSCs was developed at the Pharmaceutical Production Research Facility (PPRF, University of Calgary, Canada). The preparation of PPRF-msc6 has previously been described in detail^12^. Passage 2 (P2) hBM-MSCs (from three different donors) were thawed and inoculated into gelatin-coated (0.10 g of Type B bovine gelatin (Sigma) in 100 mL of cell culture water (Lonza, Walkersville, MD, USA)) Nunc T-flasks (Thermo Scientific, Waltham, MA, USA) at 5,000 cells/cm^2^ in growth medium (at a volume/area ratio of 0.32 mL/cm^2^). Cell cultures were incubated at 37 °C in a humidified atmosphere at 5% CO_2_. After 3 days, 50% of the medium was replaced with fresh growth medium. When the cells reached 80–90% confluence, cells were harvested by incubation with 0.05% trypsin-EDTA (Life Technologies) at 37 °C for 3–5 min. FBS-DMEM was then added to neutralize the reaction. The harvested cells were then centrifuged at 300 ***g*** for 10 min and re-suspended in fresh PPRF-msc6 and re-plated into new gelatin-coated flasks at 5,000 cells/cm^2^. At P5, after 72 hours, PPRF-msc6 was removed and the cells were washed twice with Neurobasal^®^-A medium (Life Technologies). Neurobasal-A medium was then added at the same ratio as the PPRF-msc6. Cells were placed in a humidified incubator at 37 °C and 5% CO_2_, for 24 h. Afterwards, the conditioned medium (CM) was collected as previously described^13^.

### Expansion of hBM-MSCs in dynamic (bioreactor) conditions

A DASGIP Parallel Bioreactor system (DASGIP, Julich, Germany) was used for the expansion of hBM-MSCs in dynamic conditions as previously described^51^. The bioreactors were maintained at (1) 37 °C using a heating jacket, (2) 100% dissolved oxygen (corresponding to oxygen saturation of the medium at 37 °C exposed to 21% O_2_ in the headspace), (3) a pH of 7.4, controlled by a gas mixture hooked up to oxygen, nitrogen, carbon dioxide and air tanks that was introduced into the headspace, and (4) agitated at 52 rpm.

### Preparation of microcarriers, inoculation of hBM-MSCs and collection of CM

Cytodex 3 microcarriers (GE Healthcare, Uppsala, Sweden) were used for this study and were prepared as follows. 1.0 g of microcarriers were weighed out and hydrated in 50 mL of 1× PBS (Life Technologies) in a 125 mL, pre-siliconized, Erlenmeyer flask, at room temperature overnight. To this flask 2–3 drops of Tween 80 (United States Biochemical Corporation, Cleveland, OH, USA) was added to break the surface tension and ensure proper wetting and sedimentation of the microcarriers. The microcarriers were then washed 3× with 1× PBS, and autoclaved. The microcarriers were then washed twice with our serum-free medium, and then inoculated into our 500 mL DASGIP bioreactors in 275 mL of medium for 4 h at the controlled culture conditions. hBM-MSCs were first expanded in Nunc T-flasks (Thermo Scientific) pre-coated with a 0.1% gelatin solution. Briefly, cryopreserved hBM-MSCs at passage 2 (P2) were expanded in PPRF-msc6 in static culture for two passages before inoculation into the DASGIP bioreactors. The cells were harvested using trypsin-EDTA, and then inoculated into the bioreactors at a density of 24,000 cells/mL (based on the final volume of 500 mL). The volume of the bioreactors was maintained at 325 mL for the first 24 h to increase cell attachment. After 24 h the culture volume was increased to 500 mL to bring the final microcarrier density to 2.0 g/L. The cells were cultured on the microcarriers for 72 h, after which time the bioreactors were removed from the DASGIP system, and placed in a biosafety cabinet for 10 min to allow the microcarriers to settle. The supernatant was removed from the bioreactors, and the microcarriers were washed once with 100 mL of Neurobasal-A medium. Following this, 500 mL of Neurobasal-A medium with 1% of kanamycin (Life Technologies) was added to the bioreactors and the bioreactors were placed back into the DASGIP control system for 24 h. After 24 h the bioreactors were again removed from the system, placed in a biosafety cabinet for 10 min to allow the microcarriers to settle, the supernatant was harvested and centrifuged at 300 ***g*** for 10 min to remove any cell debris. This supernatant, called the dynamic CM, was then placed at −80 °C until it was required.

### Growth of hNPCs and incubation with hBM-MSCs CM

hNPCs were isolated from the telencephalon region of a 10-week post-conception (gestational age) fetus, as we have recently described^51^. Ethical consent was approved by the Conjoint Health Research Ethics Board (CHREB), University of Calgary (ID: E-18786). So, pre-isolated and cryopreserved hNPCs were thawed and placed into a Nunc T-25 flask containing 5 mL of a serum-free medium PPRF-h2 (described in detail in^52^). After two days, cells were harvested and mechanically dissociated into a single cell suspension, and subcultured into fresh PPRF-h2. Every 4 days, T-flasks were fed by replacing 40% of the spent medium with fresh PPRF-h2. After 14–20 days of growth, hNPCs were passaged and plated onto pre-coated [(poly-D-lysine hydrobromide (100 μg/mL) and laminin (10 μg/mL) - Sigma)] 24-well plates at a density of 4.0 × 10^4^ cells per well for 5 days with the hBM-MSCs CM obtained from either the static or dynamic condition and placed in the incubator at 37 °C, 5% CO_2_, 95% air and 90% relative humidity. Neurobasal-A medium with 1% of kanamycin (Life Technologies) was used as control, as already described by our research group^53^.

### Gene expression analysis

Total RNA was extracted by using Trizol solution (Life Technologies). The cDNA synthesis was done using the iScript cDNA Synthesis Kit (BioRad, Hercules, California, USA). For quantitative gene expression analysis, the cDNA was subject to PCR amplification using SYBR Green PCR Kit (QIAGEN, Manchester, UK) on the BioRad CFX 96 Real-Time System in order to obtain the real-time detection of PCR products. The cycling conditions used in this procedure were 15 min at 95 °C, 15 s at 94 °C for denaturation, 30 s at 60 °C and 30 s at 72 °C for annealing and extension respectively. Each sample was made in triplicate for the gene of interest and HMBS was used as a housekeeping gene (see Table 1). Mean and standard error values were used for quantification, being presented the ratio between our gene of interest and HMBS.

### Transplantation of hBM-MSCs CM: Stereotaxic surgery

Eight weeks old male *Wistar-Han* rats (Charles River, Barcelona, Spain) were housed (two per cage) and maintained in a controlled environment at 22–24 °C with 55% humidity, on a 12 h light/dark cycle and fed with regular rodent’s chow and tap water *ad libitum*. Animals were handled for 1 week prior to beginning injections, in order to reduce the stress induced by the surgical procedures. All manipulations were done after approval from the *Ethical Subcommittee in Life Sciences and Health* (SECVS; ID: SECVS-008/2013, University of Minho) and from the Portuguese national authority for animal experimentation, *Direção Geral de Veterinária* (DGAV; ID: DGAV28421), and in accordance with the approved guidelines and regulations on animal care and experimentation stated in the European Union Directive 2010/63/EU. Thus, for experimental purposes, three experimental conditions (n = 5/condition) were evaluated with cerebral injection of either: 1) Neurobasal-A medium without MSC factors (Sham), 2) Static-conditioned media (CMs) or 3) Dynamic-conditioned media (CMd). Adult rats were anesthetized with ketamine hydrochloride (150 mg/kg) plus medetomidine (0.30 mg/kg). Using a stereotaxic system (Stoelting, Wood Dale, IL, USA) and a Hamilton syringe (0.5 μl Hamilton Bonaduz AG, Bondauz, CH) all injections made in these three groups were bilateral according to previously determined coordinates (Anterior/Posterior (AP) = 3.5 mm; Dorsal/Ventral (DV) = 3.5−3.1 mm; Lateral (L) = 2.0 mm)^13^,^54^. The volume injected per DG was 0.5 μl with a rate of injection of 0.25 μl/min. Two minutes were allowed after each injection in order to avoid any backflow up the needle tract. Sham group was only injected with 0.5 μl of Neurobasal-A medium; CMs and CMd groups were injected with 0.5 μl of hBM-MSC CM from the respective growth conditions. At the end, the animals were sutured and then injected with 100 μl of anti-sedating (Orion Pharma, Espoo, FIN) in order to recover from surgical procedure.

### Immunostaining - *In Vitro* Immunostaining of hNPCs

hNPCs were fixed, washed, and blocked as previously described^13^, being followed by a 1 h incubation (at 37 °C) with primary antibodies: nestin (1:200, Millipore, Billerica, MA, USA) to detect neural progenitors, rabbit anti-doublecortin (DCX; 1:500, Abcam, Cambridge, MA, USA) to detect immature neurons, mouse anti-rat microtubule associated protein-2 (MAP-2; 1:500, Sigma) and neuronal nuclei (NeuN; 1:100, Millipore) to detect mature neurons. Secondary antibodies namely, Alexa Fluor 488 *goat anti-rabbit* immunoglobulin G (IgG, Life Technologies) and Alexa Fluor 594 *goat anti-mouse* immunoglobulin G (IgG, Life Technologies) were used for 1 h at 37 °C and then 10 min with *4-6-diamidino-2-phenylindole-dihydrochloride* (DAPI; Life Technologies). Samples were observed under an Olympus BX-61 Fluorescence Microscope (Olympus, Hamburg, Germany). For this purpose, three coverslips and ten representative fields per condition were chosen and analyzed. In order to normalize the data between the different sets, the results are presented in percentage of cells. This was calculated by counting the cells positive for NeuN/MAP-2/DCX markers, and dividing this value by the total number of cells/field (DAPI-positive cells; n = 3).

### *In Vivo* Immunostaining

Regarding the *in vivo* experiment, frozen coronal sections were obtained by cryostat with a thickness of 20 μm. Sections were permeabilized and washed as previously described^13^. For Ki-67 staining antigen retrieval was performed with citrate buffer treatment (15–20 min on microwave). Sections were then washed in 1× PBS and blocked in 10% FCS/PBS-T for 2 h. Primary antibodies namely, Ki-67 (1:100, Millipore, Billerica, MA, USA) for proliferation, DCX (1:300, Abcam) for immature neurons, and rabbit anti-rat glial fibrillary acid protein (GFAP; 1:200, Dako, Carpinteria, CA, USA) for astrocyte detection were incubated overnight at 4 °C, being followed by secondary antibodies incubation: Alexa Fluor 568 *goat anti-mouse* immunoglobulin G (IgG, Life Technologies), Alexa Fluor 488 *goat anti-rabbit* immunoglobulin G (IgG, Life Technologies) for 2 h and then 10 min with *4-6-diamidino-2-phenylindole-dihydrochloride* (DAPI; Life Technologies). Images were obtained with a confocal microscope (Olympus FV1000) using the software FV10-ASW 2.0c (Olympus), presenting the hippocampal DG (five sections per animal were analyzed for n = 5/group). All granular cell layer (GCL) area was defined and the cell counts (for each marker) were made in this area.

### Proteomics – Mass Spectrometry and SWATH Acquisition

hBM-MSCs CM was processed as previously described^51^. Briefly, the CM was concentrated using a 5 kDa cut-off concentrator (Vivaspin^TM^, GE Healthcare) and the secreted proteins were precipitated from the concentrated medium using the TCA-acetone procedure^55^. One hundred micrograms of protein per sample were subjected to liquid digestion with trypsin (2 μg of trypsin/sample, overnight at 37 °C)^51^,^56^, and the formed peptides were de-salt using OMIX tips with C18 stationary phase (Agilent Technologies) before LC-MS/MS.

Samples were quantified using the 2D-Quant Kit (GE Healthcare) and 100 μg of each sample were subjected to liquid digestion. Briefly, 4 μL of 50 mM TCEP was added to 45 μL of sample, followed by ultra-sonication for 2 min. Next, 2 μL of 600 mM MMTS was added and samples were left to react for 10 min, at room temperature. TEAB was then added to bring the final volume of each sample to 100 μL, and the samples were digested with trypsin overnight (2 μg trypsin/sample), at 37 °C, with swirling at 650 rpm. Reactions were stopped by the addition of 2 μL of formic acid (FA, Sigma) and the peptides were dried by rotary evaporation under vacuum.

LC-MS/MS analysis was performed as previously detailed^51^,^56^ on a Triple TOF^TM^ 5600 System (SCIEX, Framingham, MA, USA) by performing both information-dependent acquisition (IDA) and SWATH acquisition on the same sample. Peptides were resolved by liquid chromatography (nanoLC Ultra 2D, Eksigent, Redwood City, CA, USA) on a Halo Fused-Core^TM^ C18 reverse phase column (300 μm × 15 cm, 2.7 μm particles, 90 Å, Eksigent) at 5 μL/min using an acetonitrile gradient in 0.1% FA (2% to 35% ACN, in a linear gradient for 25 min).

From the IDA experiments, a specific library of precursor masses and fragment ions was created and used for subsequent SWATH processing. The library was obtained by searching against the *human* and *bovine* species from UniProt database using the Protein Pilot^TM^ software (v4.5, AB SCIEX). The SWATH Quantitative information was extracted from the SWATH-MS data using the SWATH^TM^ processing plug-in for PeakView^TM^ (v2.0.01, AB SCIEX). Peak areas were extracted (in an extracted-ion chromatogram (XIC) window of 4 min) for up to 5 target fragment ions (automatically selected) of up to 15 peptides (selected based on a FDR lower that 1%) per protein. The levels of the human proteins were estimated by summing all the transitions from all the peptides for a given protein (an adaptation of^57^) and were normalized to the total intensity of the sample.

### Bioplex-Luminex analysis

hBM-MSCs CM was firstly concentrated using a 5 kDa cut-off filters (Vivaspin, GE Healthcare) according to manufacturer’s guidelines as previously described. Then, a targeted proteomic analysis of specific molecules such as BDNF, VEGF, NGF, and IGF-1 was performed using a Bioplex-Luminex assay. Samples were analyzed in a MAGPIX Luminex’s xMAP^®^ instrument (Luminex, Texas, USA), and the protein concentration was calculated/obtained using Bioplex Manager^TM^ 6.1 Software. The results were then normalized to cell density (i.e. pg (of each factor)) per 10.000 cells in each condition, respectively.

### Cell Counts and Normalization of CM Proteins

Cell counts from the respective conditions were performed on the day of CM harvest. For static culture, flasks were enzymatically dissociated with Trypsin-EDTA (Life Technologies) for 4–5 min at 37 °C. Trypsin activity was stopped with 10% FBS-DMEM (Life Technologies), and the suspended cells were collected and placed in a 15 mL conical tube. Cells were centrifuged and ressuspended in a known volume. Duplicate cell counts were performed using trypan blue exclusion. Based on the cell counts, cell density at time of CM harvest was determined. The amount of pg/cell was calculated using the following equation (1):


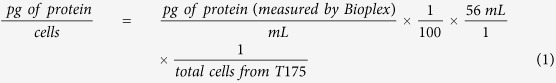


where, the pg/mL measured by Bioplex is 100× concentrated from the harvested CM. From each T175, 56 mL of CM was recovered.

For dynamic culture, a 3.0 mL representative sample of the culture (containing CM, cells and microcarriers) was harvested and placed into a 15 mL conical tube. The microcarriers were allowed to settle for 5 min, and the CM was removed and discarded. The microcarrier pellet was washed 1.0 mL 1× PBS and allowed to settle for 5 min. After settling, the supernatant was removed and the microcarriers were ressuspended in 1 mL of 1% crystal violet in 0.1 M citric acid placed in the incubator at 37 °C for 1 h. After 1 h, the microcarrier suspension was agitated vigorously 30 times with a P1000 pipette, set to 1000 mL, to burst the cells and release the nuclei. The stained nuclei were counted on a hemacytometer, and the cell density at time of CM harvest was determined. The amount of pg/cell was calculated using the following the equation (2):


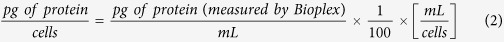


where, the pg/mL measured by Bioplex is 100× concentrated from the harvested CM.

### miRNA isolation and Real-time PCR

miRNAs were purified from conditioned medium using mirVana™ miRNA Isolation Kit, with phenol (ThermoFischer Scientific, Grand Island, NY, USA), according to manufaturer’s instructions. RNA samples (50 ng) were treated with qScript™ microRNA cDNA Synthesis Kit (Quanta Biosciences, Gaithersburg, MD, USA) to generate cDNA. qRT-PCR was performed using PerfeCTa microRNA assay for miR-16-5p (Quanta Biosciences) and PerfeCTa Universal PCR primer (Quanta Biosciences). The cycling conditions used in this procedure were 15 min at 95 °C, 15 s at 95 °C for denaturation, 20 s at 60 °C and 30 s at 72 °C for annealing and extension respectively. Samples were analyzed using 5x HOT FIREPol^®^ EvaGreen^®^ qPCR Mix Plus (ROX, Solis BioDyne, Tartu, Estonia), in an AB7500 fast Real Time PCR system (Applied Biosystems). SNORD44 small nucleolar RNA was used as internal reference. Results are presented as mean relative expression (2^ΔΔCT^).

### Statistical Analysis

Statistical evaluation for protein quantifications were performed by Student’s t-test for single comparisons, and one-way analysis of variance (ANOVA) with Bonferroni test for multiple comparisons (e.g. *in vitro* and *in vivo* experiments) through SPSS statistic program (version 22; IBM Co., USA). Data is presented as mean ± SEM. Significance value was set at p < 0.05.

## Additional Information

**How to cite this article**: Teixeira, F. G. *et al*. Modulation of the Mesenchymal Stem Cell Secretome Using Computer-Controlled Bioreactors: Impact on Neuronal Cell Proliferation, Survival and Differentiation. *Sci. Rep*. **6**, 27791; doi: 10.1038/srep27791 (2016).

## Figures and Tables

**Table 1:** PCR primers used to detect gene expression in hNPCs-differentiated cells.

Gene	Primer sequence	Product size (bp)	NCBI accession number
NOTCH1	Forward 5′-GATGGC ACGACGCCACTGAT-3′Reverse 5′-GGGGTGTCTCCTCCCTGTTGTT-3′	214	NM_017617.3
HMBS	Forward 5′-TCGGGGAAACCTCAACACC-3′Reverse 5′- CCTGGCCCACAGCATACAT-3′	150	NG_008093.1
